# Circular RNA Expression Profiles in Crimean–Congo Hemorrhagic Fever: Insights into Disease Pathogenesis and Fatal Outcomes

**DOI:** 10.3390/ijms27156738

**Published:** 2026-07-28

**Authors:** Serdal Arslan, Derya Yetkin, Adnan Selim Kimyon, Fatma Söylemez, Gülhan Temel, Mehmet Bakır

**Affiliations:** 1 Department of Medical Biology, Faculty of Medicine, Mersin University, 33343 Mersin, Turkey; fsoylemez@mersin.edu.tr; 2 Advance Technology Education Research and Application Centre, Mersin University, 33343 Mersin, Turkey; deryayetkin84@gmail.com; 3 Vocational School of Health Services, Near East University, 99138 Nicosia, Cyprus; selimkimyon@gmail.com; 4 Department of Biostatistics and Medical Informatics, Faculty of Medicine, Mersin University, 33343 Mersin, Turkey; gulhanorekici@mersin.edu.tr; 5 Department of Infectious Disease, Medicana Hospital, 58000 Sivas, Turkey; mehmetbakir57@yahoo.com

**Keywords:** Crimean–Congo hemorrhagic fever, circular RNA, CCHF, circRNA expression level

## Abstract

Crimean–Congo hemorrhagic fever (CCHF) is a severe tick-borne viral disease with high mortality rates and no approved specific antiviral therapy or vaccine. Circular RNAs (circRNAs) are emerging regulators of gene expression and have been implicated in various infectious and inflammatory diseases; however, their role in CCHF remains unknown. In this study, we investigated circRNA expression profiles in peripheral blood samples from CCHF patients and healthy controls. Total RNA was isolated, enriched for circRNAs using RNase R treatment, and analyzed with the Arraystar Human circRNA Array V2 platform. Differential expression analysis was performed using the limma package in R, followed by RT-PCR validation and bioinformatic analyses. Compared with healthy controls, 15 circRNAs were significantly upregulated and 264 were significantly downregulated in CCHF patients. The most highly upregulated circRNAs included hsa_circ_011083, hsa_circ_092556, hsa_circ_001766, hsa_circ_001569, and hsa_circ_027934, whereas hsa_circ_405788, hsa_circ_104982, hsa_circ_002008, hsa_circ_406768, and hsa_circ_037139 were among the most downregulated. Additionally, 641 and 455 differentially expressed circRNAs were identified in fatal–control and fatal–nonfatal comparisons, respectively. Functional enrichment analyses revealed associations with immune response, inflammatory signaling, and host–virus interaction pathways. To our knowledge, this is the first study to characterize circRNA expression patterns in CCHF, providing novel insights into disease pathogenesis and identifying potential biomarkers associated with disease severity and mortality.

## 1. Introduction

The Crimean–Congo hemorrhagic fever virus was first reported in 1944 during a case series of a febrile disease that had features of hemorrhagic fever in Soviet soldiers stationed in Crimea, while the etiologic agent was later isolated from a febrile individual from the Congo. Currently, it is known that the distribution of CCHF is extensive and includes many regions across Asia, Europe, the Middle East, and Africa. Recently, the WHO added the CCHF virus (CCHFV) to the priority pathogens list [1].

CCHFV is an enveloped, negative-sense RNA virus with a diameter of approximately 100 nm and a tripartite genome consisting of the small (S), medium (M), and large (L) segments. The L segment encodes the RNA-dependent RNA polymerase, the M segment encodes the glycoproteins Gn and Gc, and the S segment encodes the nucleocapsid protein. CCHFV, a member of the genus *Nairovirus* within the family Bunyaviridae, enters host cells via receptor-mediated endocytosis. After entry, replication occurs in the cytoplasm, starting with complement RNA (cRNA) synthesis [2]. Viral proteins and RNA then combine to form nucleocapsids that are processed through the endoplasmic reticulum and Golgi apparatus before the new virions are released from the host cell [3].

CCHFV is primarily transmitted through ticks of the genus *Hyalomma*. Transmission occurs via tick bites or direct contact with the blood, body fluids, and tissues of infected humans or animals. The incubation period varies depending on the route of exposure; it ranges from 1 to 9 days after a tick bite, and 5–13 days in case of contact with contaminated blood or tissues [4]. In clinical terms, CCHF generally passes through four phases: incubation, pre-haemorrhagic, haemorrhagic, and convalescence. In the primary stage of viral multiplication (around the first week), thrombocytopenia and leukopenia occur, and the concentrations of liver enzymes become elevated. Symptoms commonly include weakness, nausea, fever, headaches, chills, and muscle pain. After entering the blood, the virus spreads to several body systems, such as the liver, kidneys, and brain. Despite the differences in pathogenesis for each person with CCHF, the exact mechanism remains unknown [5].

The epigenetic processes act as critical modulators of transcription and translation of genes in eukaryotes, where DNA, histone proteins, and ncRNAs are among the major targets [6]. The role of ncRNAs in epigenetic gene regulation is especially significant. Circular RNAs (circRNAs), a recently characterized class of ncRNAs, are single-stranded, covalently closed molecules that demonstrate cell type-, tissue type-, and developmental stage-specific expression patterns. CircRNAs regulate gene expression in various ways, including acting as microRNA (miRNA) sponges, influencing alternative splicing, modulating protein transport, and, in some cases, encoding proteins. They also contribute to ribosomal RNA (rRNA) and transfer RNA (tRNA) biogenesis [7]. Depending on sequence length, a single circRNA can bind 4–14 miRNAs, while a single miRNA is estimated to regulate the expression of approximately 200 genes. Consequently, dysregulation of circRNA expression may profoundly alter cellular transcriptomes, underscoring its relevance to disease pathogenesis [8].

CircRNAs have been shown to exhibit altered expression under pathological conditions and are strongly associated with the development of various human diseases [9]. Accordingly, modulation of circRNA expression is increasingly considered a potential therapeutic strategy. Cellular immune factors also contribute to circRNA biogenesis and may suppress viral infections. Both host- and virus-derived circRNAs interact with double-stranded RNA (dsRNA)-binding antiviral proteins, thereby influencing immune responses during host–virus interactions [10]. Similarly, cellular miRNAs play critical roles in host–virus dynamics by regulating genes involved in immunity and viral pathogenesis [11]. Viruses can also exploit host miRNAs to support their replication and survival. In infected cells, circRNAs may inhibit viral replication by suppressing miRNA activity, whereas viral miRNAs can modulate host cellular processes, including apoptosis and tumor-related pathways [12]. In some cases, virus-encoded miRNAs suppress viral gene expression, thereby protecting infected cells from immune surveillance. CircRNAs can enhance antiviral immune responses by antagonizing the effects of viral miRNAs. Accordingly, modulating miRNA–circRNA interactions offers a promising therapeutic strategy against viral infections. Further exploration of CCHFV-specific circRNA–miRNA interactions could reveal novel therapeutic targets for this priority pathogen [13,14]. Considering the well-established role of circular RNAs (circRNAs) in modulating gene expression via miRNA sponging, a comprehensive characterization of the circRNA–miRNA–mRNA regulatory networks during Crimean–Congo hemorrhagic fever virus infection is essential for elucidating disease progression and identifying novel therapeutic targets.

To date, circRNA expression in CCHF has not been systematically investigated. The present study, for the first time, identifies circRNAs implicated in the pathogenesis of CCHF using microarray analysis. Furthermore, we identified circRNAs potentially associated with disease severity and fatal outcomes in patients with CCHF.

## 2. Results

**Figure 1:**
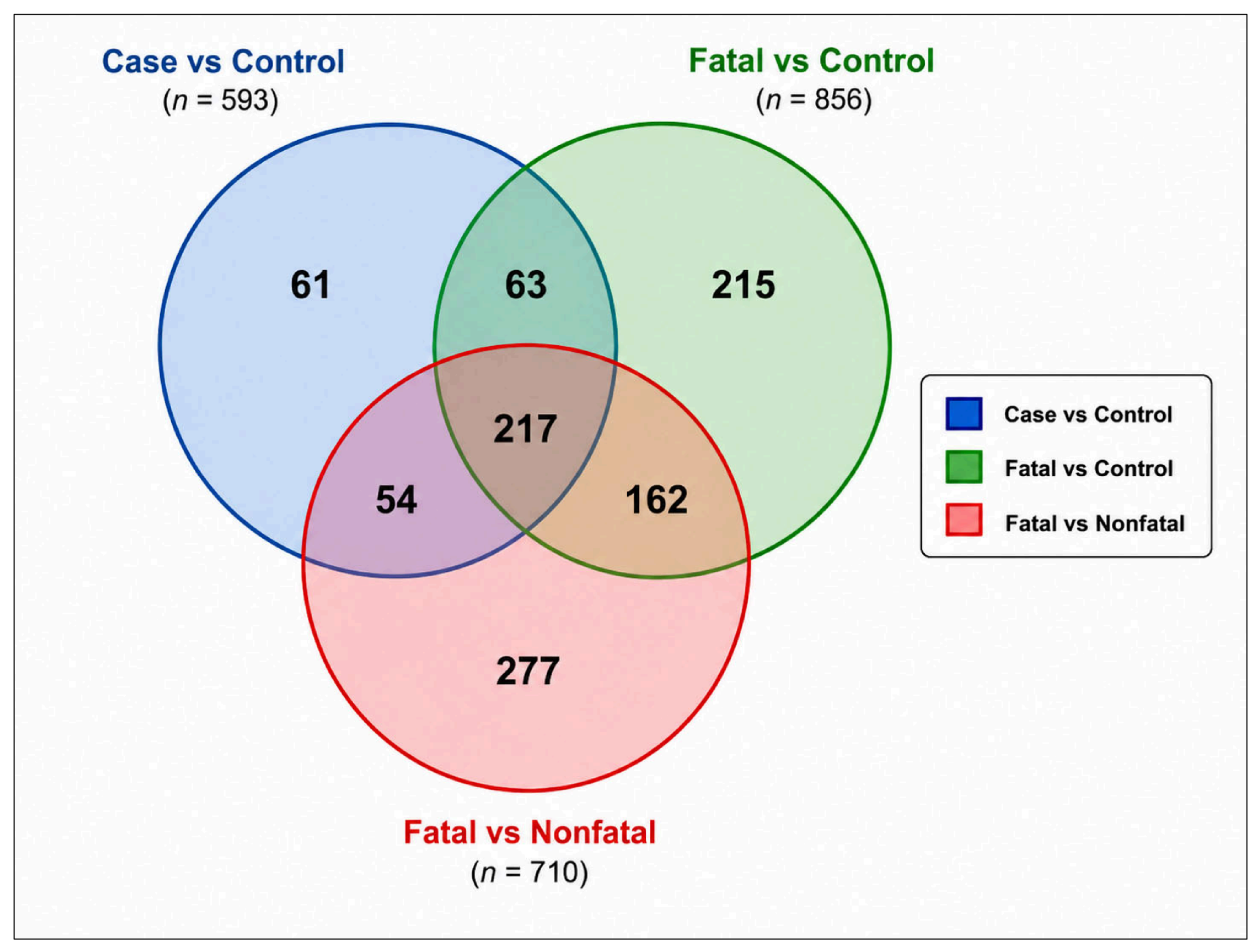
Venn diagram showing the overlap of differentially expressed circRNAs among the Case vs. Control, Fatal vs. Control and Fatal vs. Nonfatal comparisons.

CircRNA microarray analysis was conducted on 14 samples, comprising 9 from patients with Crimean–Congo hemorrhagic fever and 5 from healthy controls. Among the nine patients, four were classified as fatal CCHF cases, while the remaining five were non-fatal. No statistically significant differences were observed between the groups in terms of age or gender distribution (*p* > 0.05). The patient cohort consisted of nine individuals (5 females and 4 males; mean age: 41 years), while the control group included five individuals (3 females and 2 males; mean age: 39 years). Venn diagram analysis revealed 61, 215, and 277 uniquely expressed circRNAs in the Case vs. Control, Fatal vs. Control, and Fatal vs. Nonfatal comparisons, respectively. Furthermore, 63 circRNAs were shared between the Case vs. Control and Fatal vs. Control comparisons, 54 between the Case vs. Control and Fatal vs. Nonfatal comparisons, and 162 between the Fatal vs. Control and Fatal vs. Nonfatal comparisons. Notably, 217 circRNAs were common to all three comparisons (Figure 1). The complete list of the 217 circRNAs commonly identified across all three comparisons is presented in Supplementary File S1.

**Figure 2:**
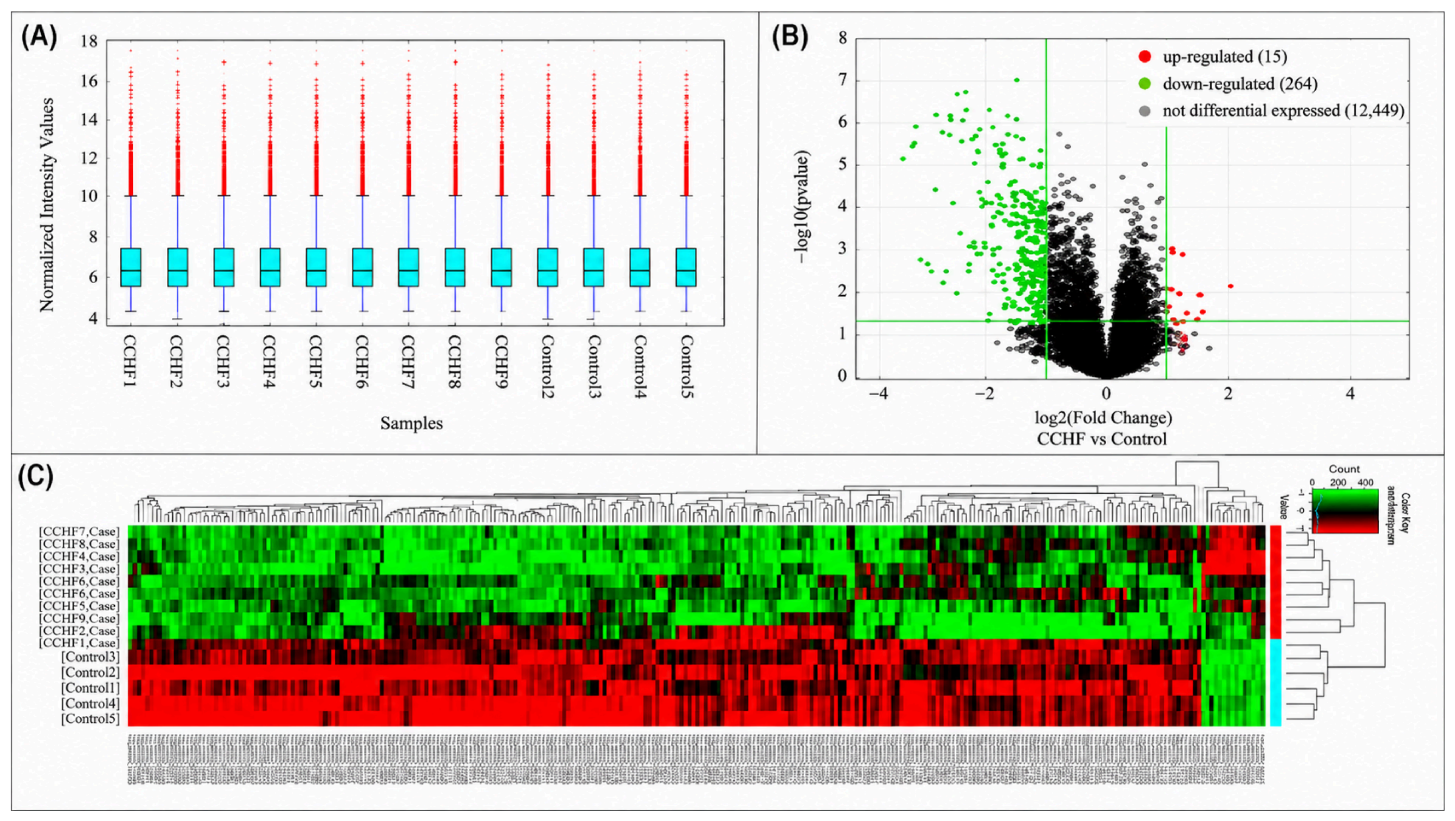
(**A**) Box plots showing the distribution of normalized intensity values in CCHF patient and healthy control samples. (**B**) Microarray results of statistically significant DE circRNAs volcano plots: upregulated (red), downregulated (green), and no differential expression (gray). (**C**) Unsupervised hierarchical clustering heatmap of significantly differentially expressed circRNAs between CCHF patients and healthy controls. The color scale varies from red to green; red suggests upregulated circRNAs, and downregulated circRNAs are shown in green.

The distribution of normalized intensity values across all samples is presented in the box plot of the microarray data (Figure 2A). Differential expression analysis identified 15 significantly upregulated circRNAs and 264 significantly downregulated circRNAs in CCHF patients relative to healthy controls, with 12,449 circRNAs exhibiting no significant changes in expression. The results of the differential expression analysis are visualized by a volcano plot (Figure 2B) and a heatmap of significantly altered circRNAs (Figure 2C).

The *p*-values, false discovery rates (FDRs), fold changes, and predicted miRNA targets of the 10 upregulated and 10 downregulated circRNAs identified between the patient and control groups are summarized in Table 1. The five most upregulated circRNAs in CCHF patients relative to healthy controls were hsa_circ_011083, hsa_circ_092556, hsa_circ_001766, hsa_circ_001569, and hsa_circ_027934. Conversely, the five most downregulated circRNAs comprised hsa_circ_405788, hsa_circ_104982, hsa_circ_002008, hsa_circ_406768, and hsa_circ_037139. Detailed statistical and functional data for these ten significantly dysregulated circRNAs are presented in Table 1, whereas the complete list of all upregulated and downregulated circRNAs is provided in Supplementary File S2.

In this study, circRNA expression levels in patients with CCHF were compared between fatal cases and controls. A total of 641 circRNAs were found to be significantly differentially expressed in fatal cases compared with controls. Among these, 148 circRNAs were upregulated, whereas 493 were downregulated (Supplementary File S3). Table 2 shows that 10 circRNAs were upregulated and 10 were downregulated in fatal cases relative to controls. In fatal CCHF patients, hsa_circ_011083 was the most significantly upregulated circRNA (FC = 12.95, *p* = 0.04), followed by hsa_circ_011084 and hsa_circ_102485. Conversely, hsa_circ_037139, hsa_circ_403874, and hsa_circ_404163 were the most significantly downregulated circRNAs. Detailed information on fold changes, statistical significance, and predicted miRNA targets is provided in Table 2.

Differential expression analysis between fatal and non-fatal CCHF patients revealed a distinct circRNA profile associated with disease severity. Table 3 summarizes the *p*-values, false discovery rates (FDRs), fold changes, and predicted miRNA targets of the 10 significantly dysregulated circRNAs identified between fatal and non-fatal CCHF patients, while the complete list of significantly dysregulated circRNAs is presented in Supplementary File S4. Among the upregulated circRNAs, hsa_circ_007738 (FC = 11.49, *p* = 0.03) and hsa_circ_000250 (FC = 6.37, *p* = 0.03) showed the highest expression differences. Among the upregulated circRNAs, hsa_circ_007738 and hsa_circ_000250 showed the greatest expression differences, whereas hsa_circ_403874 and hsa_circ_001937 were the most significantly downregulated. Detailed information regarding their predicted miRNA targets is presented in Table 3.

**Figure 3:**
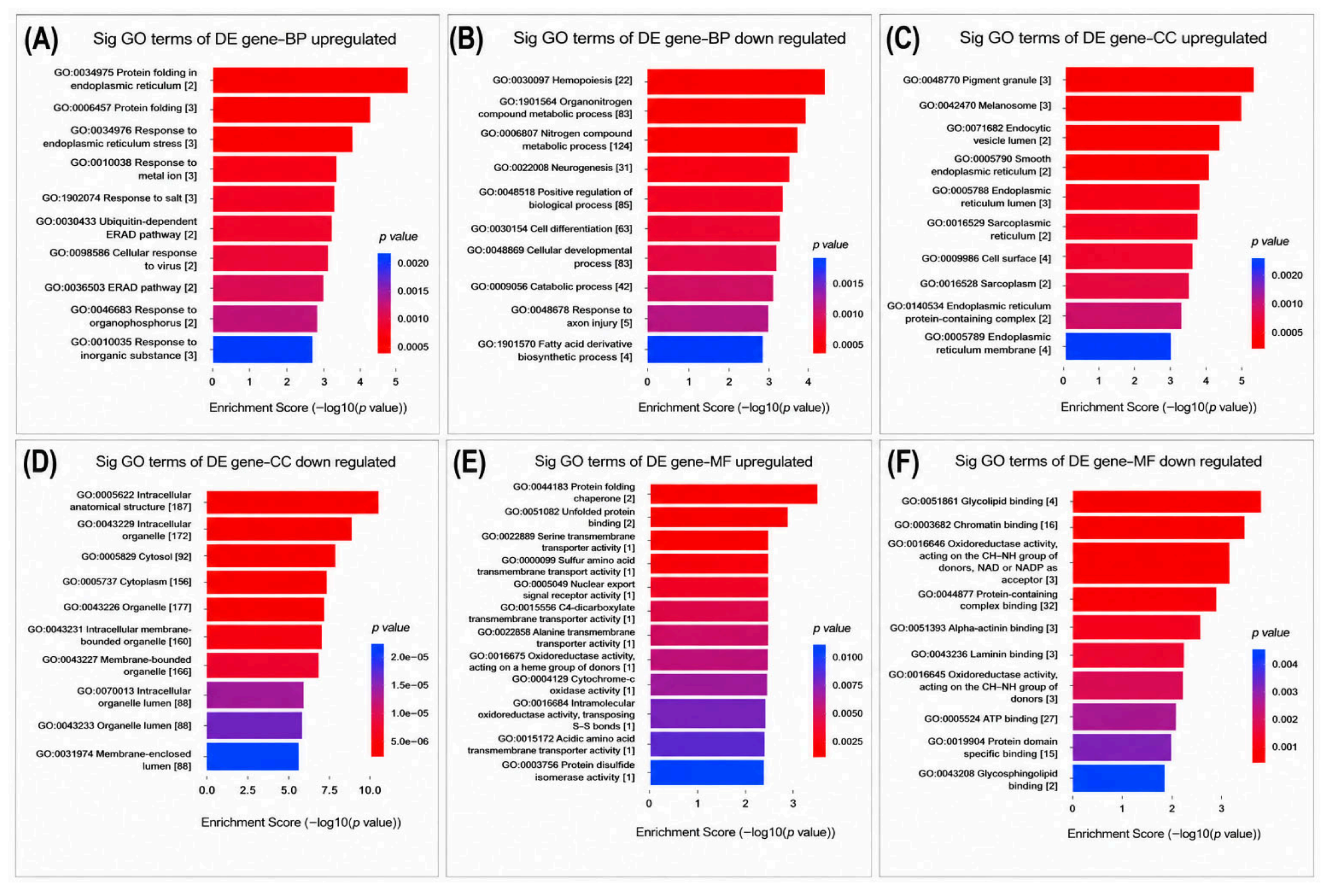
Significant Gene Ontology of upregulated and downregulated circRNAs: Biological process (BP), Cellular component (CC), Molecular function (MF). (**A**) Case vs. control upregulated, (**B**) Case vs. control downregulated, (**C**) Fatal vs. control upregulated, (**D**) Fatal vs. control downregulated, (**E**) Fatal vs. nonfatal upregulated, and (**F**) Fatal vs. nonfatal downregulated.

Gene Ontology (GO) enrichment analysis demonstrated that both upregulated and downregulated circRNAs were significantly associated with diverse biological processes, cellular components, and molecular functions. Upregulated circRNAs were primarily enriched in processes related to endoplasmic reticulum stress, protein folding, and cellular responses to viral infection, with cellular components predominantly localized to the endoplasmic reticulum, cytosol, and vesicle lumen. Molecular functions were mainly associated with oxidoreductase activity and protein binding. In contrast, downregulated circRNAs were enriched in biological processes including metabolic regulation, cellular response to toxic stimuli, and cell differentiation. These circRNAs were mainly associated with intracellular organelles and membrane-bound compartments, while their molecular functions included protein binding, ATP binding, and oxidoreductase activity (Figure 3).

**Figure 4:**
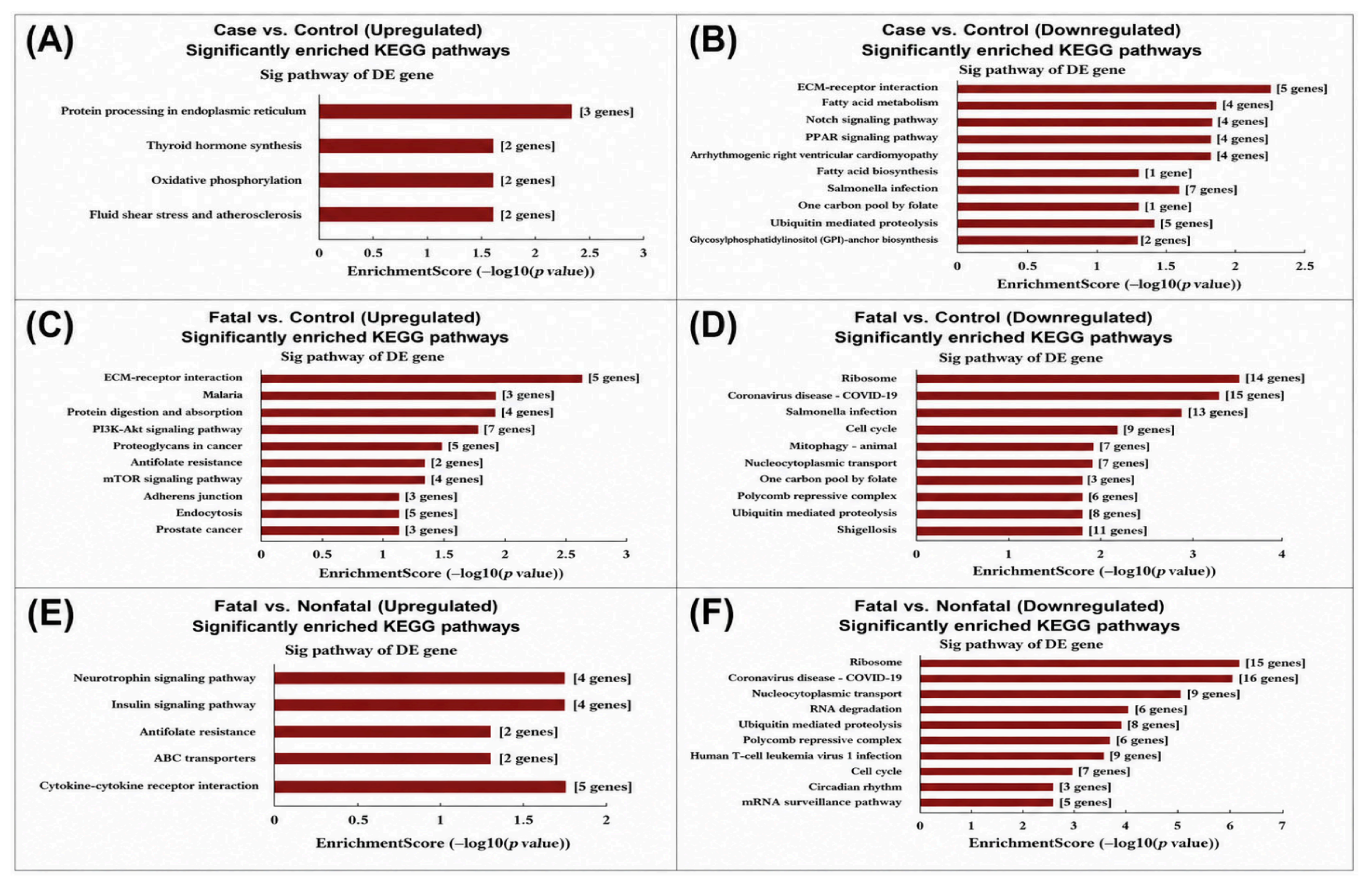
Significant KEGG pathways of statistically significantly expressed upregulated and downregulated circRNAs. (**A**) Case vs. control upregulated, (**B**) Case vs. control downregulated, (**C**) Fatal vs. control upregulated, (**D**) Fatal vs. control downregulated, (**E**) Fatal vs. nonfatal upregulated, and (**F**) Fatal vs. nonfatal downregulated.

KEGG pathway enrichment analysis revealed distinct functional patterns across case–control, fatal–control, and fatal–nonfatal comparisons (Figure 4). Upregulated genes were consistently enriched in immune- and signaling-related pathways, including cytokine signaling, Notch, and PI3K–Akt pathways, as well as metabolic processes such as fatty acid metabolism. In contrast, downregulated genes were primarily associated with oxidative phosphorylation, RNA processing, protein degradation, and cell cycle regulation.

**Figure 5:**
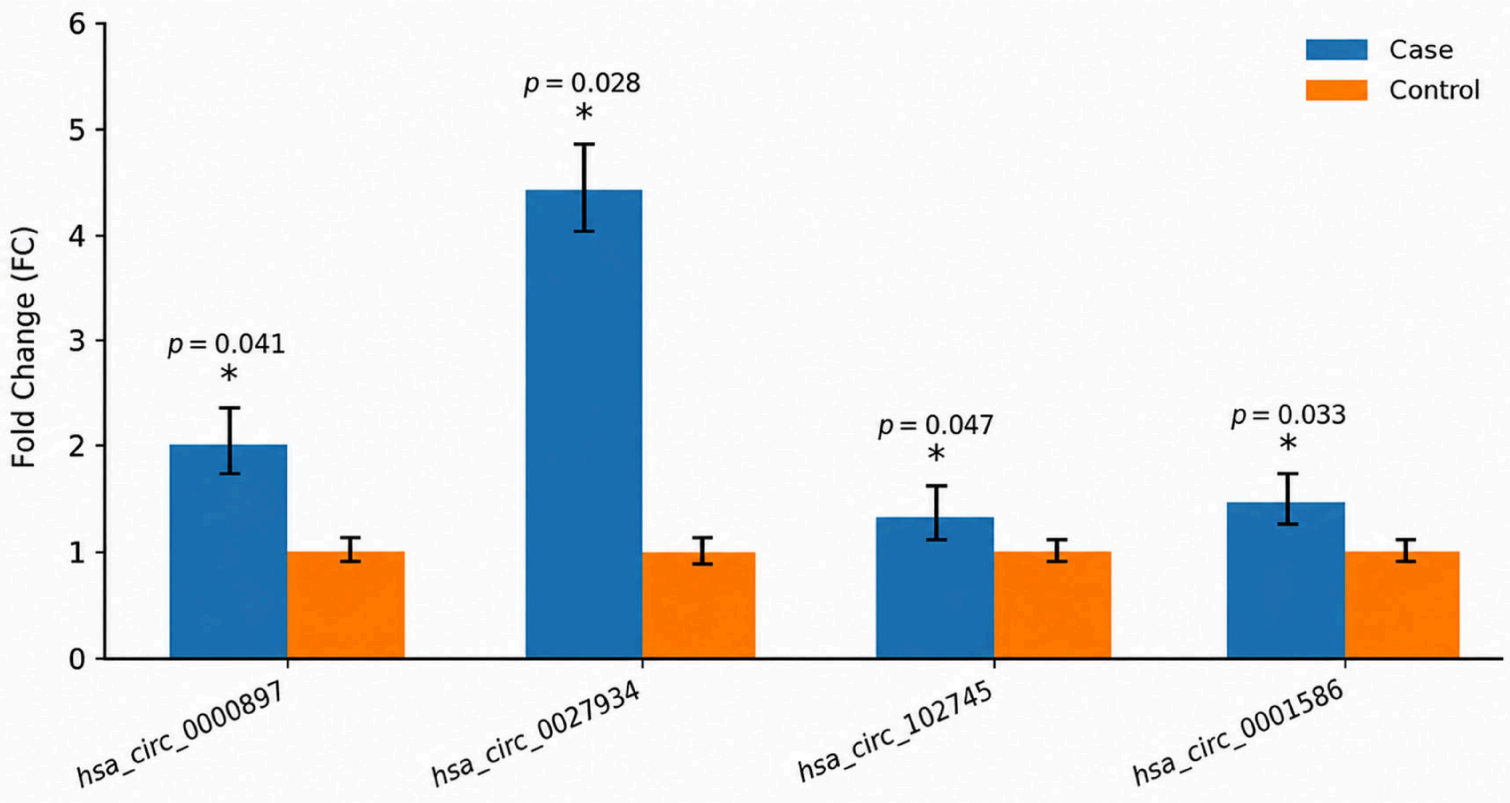
RT-qPCR validation of four significantly upregulated circRNAs identified in the case group compared with the control group. *: Indicates statistical significance (*p* < 0.05).

To validate the microarray results, the expression levels of selected circRNAs were quantified by qRT-PCR. Compared with the control group, hsa_circ_0000897 expression was increased in the case group (fold change = 2.02; *p* = 0.04). Similarly, hsa_circ_0027934 showed the highest increase in expression (fold change = 4.42; *p* = 0.02), while hsa_circ_102745 and hsa_circ_0001586 exhibited slightly elevated levels (fold changes = 1.27; *p* = 0.04 and fold changes = 1.46; *p* = 0.03, respectively) (Figure 5).

## 3. Discussion

Crimean–Congo hemorrhagic fever is a severe disease characterized by high case-fatality rates and posing significant public health threats in endemic regions. While substantial knowledge exists on the viral pathogenesis of Crimean–Congo hemorrhagic fever, the contributions of epigenetic mechanisms to disease progression remain largely underexplored [15]. In our previous studies, we identified important miRNAs and lncRNAs in the pathogenesis of CCHF using the microarray method [16,17]. Few studies have explored circRNAs’ roles in viral infection pathogenesis [18], and, to date, none have investigated them in CCHF. Recent studies suggest that circRNAs may act as therapeutic targets in viral infections due to their capacity to function as miRNA sponges and regulators of gene expression [19]. Although the disease-related roles of many circRNAs identified to date remain unknown, their significance in CCHF patients offers key insights into their functions. We filled an important epigenetic gap by detecting circRNA microarray gene expression in CCHF patients.

A prominent finding of our study was the coordinated upregulation of several circRNAs potentially involved in immune regulation, inflammatory signaling, antiviral defense, and host–virus interactions. Among these, hsa_circ_011083 and hsa_circ_092556 exhibited the highest expression levels in CCHF patients. Although the biological functions of hsa_circ_011083 remain unknown, bioinformatic analysis predicted interactions with five miRNAs, including hsa-miR-148a-5p. Notably, our previous study demonstrated significant downregulation of miR-148a in CCHF patients [16]. Since miR-148a regulates key inflammatory mediators such as IκB-β, NF-κB, IL-6, TNF-α, IL-12, and IFN-β [20], the inverse expression pattern observed between hsa_circ_011083 and miR-148a supports a potential ceRNA-mediated mechanism through which circRNA dysregulation may contribute to excessive inflammatory responses during CCHFV infection [21]. Similarly, hsa_circ_092556 was predicted to interact with members of the miR-103 family, particularly miR-103a-1-5p and miR-103a-2-5p, which are known regulators of immune responses and infectious disease progression [22]. Beyond these highly expressed circRNAs, other upregulated molecules also converged on pathways associated with immune activation and cellular adaptation to infection. For example, hsa_circ_001569 has been linked to PI3K–AKT and Wnt/β-catenin signaling pathways [23,24], both of which play important roles in immune cell activation, cellular stress adaptation, and tissue responses to injury. Likewise, hsa_circ_0000479 has been associated with immune regulation and antiviral responses through the hsa_circ_0000479/miR-149-5p/RIG-I/IL-6 axis [25], suggesting a potential role in innate antiviral defense mechanisms during CCHFV infection. Although hsa_circ_060455, hsa_circ_102745, hsa_circ_000897, hsa_circ_027934, and hsa_circ_001766 remain largely uncharacterized, their predicted interactions with miRNAs involved in inflammation, cellular stress responses, and innate immune signaling further support the biological relevance of these findings. For instance, hsa_circ_060455 was predicted to interact with miR-599 and miR-1256, whereas hsa_circ_102745 may regulate miR-147b-5p, a known negative regulator of Toll-like receptor-mediated NF-κB activation [26]. Rather than reflecting independent expression changes, the coordinated elevation of these circRNAs points to the activation of interconnected regulatory circuits that may modulate immune function, inflammation, and antiviral responses in CCHF.

Conversely, numerous circRNAs were markedly downregulated in CCHF patients, indicating a potential disruption of circRNA-dependent regulatory networks during infection. Several of these molecules, including hsa_circ_405788, hsa_circ_104982, hsa_circ_002008, and hsa_circ_037139, exhibited marked fold changes and were predicted to interact with miRNAs involved in inflammation, metabolism, cellular stress responses, and immune regulation, including members of the miR-103 family [27]. These findings suggest that reduced expression of specific circRNAs may increase the availability of immune-related miRNAs, thereby enhancing post-transcriptional repression of genes involved in host defense and cellular adaptation. Similarly, hsa_circ_404686 and hsa_circ_401844 were significantly downregulated in CCHF patients. Although hsa_circ_404686 has previously been proposed as a diagnostic biomarker in papillary thyroid microcarcinoma [28], its biological role remains poorly understood. More importantly, hsa_circ_401844 is predicted to interact with pro-inflammatory miRNAs such as miR-942-5p, suggesting that its reduced expression may contribute to dysregulated inflammatory signaling and impaired immune homeostasis during CCHFV infection. Additional downregulated circRNAs, including hsa_circ_004183 and hsa_circ_000881, may further support the involvement of circRNA-mediated regulatory pathways in disease pathogenesis. While these circRNAs have previously been associated with non-infectious diseases [29,30], their altered expression in CCHF suggests that they may participate in broader biological processes related to cellular activation, proliferation, migration, and immune responses. The consistent reduction in multiple circRNAs linked to stress-response and signaling pathways indicates that disruption of post-transcriptional regulatory networks may be a characteristic feature of CCHF. Notably, hsa_circ_102049 (hsa_circ_0043278) and hsa_circ_102051 were among the most significantly downregulated circRNAs identified in the present study. Previous studies have demonstrated that hsa_circ_102049 can sponge miR-455-3p and regulate expression of CD80, an important immune-related molecule [31,32,33]. On the other hand, a considerable repression in circRNA genes, like hsa_circ_037139 and hsa_circ_404163, can result in an increase in the action of miRNA targets such as miR-34a-5p and miR-599. The miR-34 family of RNA molecules plays an important role in cell cycle regulation and apoptosis; therefore, their enhanced activity might cause organ injury and endothelial dysfunction observed in lethal CCHF infections [34]. Similarly, miR-599 has been associated with inflammatory signaling pathways, further supporting its potential involvement in disease progression [35]. Taken together, these findings indicate that downregulated circRNAs may contribute to CCHF pathogenesis not merely as isolated molecular alterations but as components of a broader breakdown in circRNA-miRNA regulatory networks. Disruption of circRNA–miRNA regulatory interactions may facilitate immune dysregulation, uncontrolled inflammation, impaired antiviral defense, and cellular stress responses, thereby contributing to disease severity in CCHF. Taken together, the results indicate that circRNA-associated regulatory mechanisms are involved in the host response to CCHFV and may serve as promising candidates for future diagnostic and therapeutic applications.

The circRNA expression signature in fatal and non-fatal CCHF cases suggests that circRNA-miRNA interactions may be important in determining disease severity. The upregulation of circRNAs such as hsa_circ_007738 and hsa_circ_000250 may promote inflammatory signaling by sequestering key regulatory miRNAs, while the involvement of the miR-103 family further supports a role in immune and metabolic stress responses [27]. Conversely, reduced expression of certain circRNAs, such as hsa_circ_403874 and hsa_circ_001937, may elevate the function of immune-suppressive and apoptotic miRNAs, like miR-22-5p and miR-27 families. In summary, the evidence obtained from our studies shows that fatal CCHF has an imbalance between pro-inflammatory signals and compromised immunity due to dysregulation. This situation can contribute to disease progression and poor clinical outcomes.

In the analysis conducted using GO and KEGG analysis, it can be seen that CCHF is marked by a reduction in the processes of metabolism and homeostasis together with an activation in the immune system and stress responses. High circRNA levels were found to correspond to pathways related to endoplasmic reticulum stress, cytokine signaling, and PI3K-Akt signaling. This was because there was more activation in the immune response during infection. On the other hand, low circRNA levels were found to correlate with pathways relating to oxidative phosphorylation, RNA processing, and protein synthesis.

Several limitations can be attributed to this study. Firstly, there is a lack of studies on circRNA microarrays for viral diseases, especially CCHF, which restricts our interpretation of our results in the biological context. Also, the function of the majority of these circRNAs remains unknown, and therefore, it makes it difficult to analyze their impact on CCHF pathogenesis. Second, the relatively small sample size, particularly the limited number of fatal CCHF cases, may have reduced the statistical power of the analyses and affected the generalizability of the findings. Consequently, the identified circRNA expression profiles and their potential biological significance should be interpreted with caution until they are validated in larger, independent cohorts. Another limitation of this study is that the circRNA microarray platform may not capture the complete diversity of human circRNAs. Therefore, the present findings should be interpreted with this technical limitation in mind, and further studies using complementary profiling approaches are needed to validate and expand these results. However, this was primarily due to the high cost associated with circRNA microarray analysis. Despite these limitations, the identified circRNAs provide valuable preliminary insights and a basis for future studies investigating circRNA-mediated regulatory mechanisms in viral infections.

In summary, this study represents the first systematic characterization of the differential expression of circRNAs in Crimean–Congo hemorrhagic fever, along with their miRNA sponging capacity and pathway analysis. In addition, we discovered circRNAs associated with fatal outcomes in the disease, which implies their participation in the pathophysiological process. Overall, our observations support the notion that circRNA-based regulatory circuits might play a role in the development of CCHF by coordinating immune abnormalities with metabolic disorders, thereby providing promising diagnostic markers and therapeutic candidates for the disease.

## 4. Materials and Methods

### 4.1. Study Population

This study included a total of 14 samples for circRNA microarray analysis, comprising 9 samples from patients and 5 samples from healthy controls. Four samples in the patient group were derived from individuals with fatal outcomes. The study population was recruited from the Department of Infectious Diseases and Clinical Microbiology, Sivas Cumhuriyet University Application and Research Hospital. The samples used in this work were obtained from a previously published study, in which the diagnostic criteria for patient selection were described in detail [16]. All participants provided written informed consent before sample collection. Ethical approval for the study was granted by the Mersin University Ethics Committee (Decision No: 2023/329).

### 4.2. Total RNA Isolation and Microarray Analysis

Blood samples were drawn from the patient and control groups into blood tubes for RNA stabilization, followed by extraction of total RNA using the PAXgene Blood RNA Kit (Qiagen, Hilden, Germany). RNA integrity and concentration were assessed with the Agilent 2100 Bioanalyzer (Agilent Technologies, Santa Clara, CA, USA). CircRNA enrichment was achieved using RNase R treatment (ab286929; Abcam, Cambridge, UK), followed by microarray analysis using the Arraystar Human circRNA Array V2 (8x15K, Arraystar Inc., Rockville, MD, USA). To enrich circRNA content, total RNA was treated with RNase R, followed by amplification and transcription into fluorescent cRNA using a random priming approach (Arraystar Super RNA Labeling Kit, Arraystar). Labeled cRNA samples were hybridized onto the circRNA Array V2 (8x15K, Arraystar) in an Agilent Hybridization Oven. After hybridization, slides were washed and scanned using the Agilent Scanner G2505C. Raw data were extracted using Agilent Feature Extraction software (version 11.0.1.1).

To validate the microarray results, circRNAs with the highest fold changes were selected, including hsa_circ_0000897, hsa_circ_0027934, hsa_circ_102745, and hsa_circ_0001586. Total RNA was digested with RNase R according to the manufacturer’s instructions, and cDNA was synthesized from circRNA-enriched RNA. Quantitative real-time PCR (RT-qPCR) was performed using the SYBR Green method, with hsa_circ_0000284 serving as an endogenous control. Reactions were conducted in triplicate, and relative expression levels were calculated using the 2^−ΔΔCt^ method [36]. The primer sequences used were as follows: hsa_circ_0000897: F 5′-CGA GCT GTA GAG AGG CCT G-3′, R 5′-TCC TCC TCC TCT TTG CGT TT-3′, hsa_circ_0027934: F 5′-CCT TCT CC CCT GCAC TGTAA-3′ R 5′-TCA ACT TCA TCG TCA GCT CTG-3′, hsa_circ_102745: F 5′-AAA GTC GTG ACC CAG AAC AG-3′, R 5′-CGG TTG CAT ACG TAC GGA AA-3′, hsa_circ_0001586: F 5′-GAC AAG CCG GAG ACA AAA CC-3′, R 5′-CCC CTG CTT GCT CAA ATC AA-3′.

### 4.3. Statistical and Bioinformatic Analyses

Microarray data were analyzed using the limma package in R (version 4.6.1). Raw array images were processed with Agilent Feature Extraction software, followed by quantile normalization and batch effect correction with ComBat (sva package, version 3.52.0). Normalized intensity values were visualized using box plots to assess the distribution of expression data across all samples (Figure 2A). Differentially expressed (DE) circRNAs between CCHF patients and controls were identified using volcano plot filtering (Figure 2B), and expression patterns were visualized with hierarchical clustering heatmaps (Figure 2C).

Functional enrichment was assessed using Gene Ontology (GO) analysis, covering Biological Process (BP), Cellular Component (CC), and Molecular Function (MF) categories (Figure 2). Fisher’s exact test, implemented via Bioconductor’s topGO package (Bioconductor version 3.22; topGO version 2.62.0), was used to evaluate enrichment significance, with *p* ≤ 0.05 considered statistically significant. Pathway enrichment was evaluated using the Kyoto Encyclopedia of Genes and Genomes (KEGG) pathway analysis (Figure 3). Lower *p*-values indicated stronger pathway associations (cut-off: *p* ≤ 0.05).

To predict circRNA–miRNA interactions, Arraystar’s proprietary algorithm (based on TargetScan (version 8.0) and miRanda (version 3.3a)) was applied, and detailed annotations of DE circRNAs were generated. For validation, RT-qPCR results of the four selected circRNAs were analyzed using the 2^−ΔΔCt^ method with the GeneGlobe (QIAGEN, Hilden, Germany) web tool. The expression levels of selected circRNAs were evaluated in the case and control groups using quantitative real-time PCR (qPCR). hsa_circ_0000284 was used as the housekeeping gene for normalization.

## Figures and Tables

**Table 1:** Statistically significant circRNAs between patient and control groups.

circRNA	*p*-Value	FDR	FC (abs)	Regulation	MRE1	MRE2	MRE3	MRE4	MRE5
*hsa_circRNA_011083*	<0.01	0.12	4.21	up	hsa-miR-2277-5p	hsa-miR-1285-3p	hsa-miR-148a-5p	hsa-miR-3127-5p	hsa-miR-6874-3p
*hsa_circRNA_092556*	0.02	0.27	2.99	up	hsa-miR-103a-1-5p	hsa-miR-103a-2-5p	hsa-miR-6815-3p	hsa-miR-718	hsa-miR-4778-3p
*hsa_circRNA_001766*	0.01	0.16	2.94	up	hsa-miR-877-3p	hsa-miR-6881-3p	hsa-miR-182-5p	hsa-miR-6875-3p	hsa-miR-10522-5p
*hsa_circRNA_001569*	0.01	0.16	2.85	up	hsa-miR-6728-3p	hsa-miR-3194-3p	hsa-miR-1471	hsa-miR-5691	hsa-miR-4665-3p
*hsa_circRNA_027934*	0.04	0.32	2.80	up	hsa-miR-4659a-3p	hsa-miR-3163	hsa-miR-4659b-3p	hsa-miR-4778-3p	hsa-miR-4668-5p
*hsa_circRNA_001586*	0.02	0.27	2.45	up	hsa-miR-619-5p	hsa-miR-4775	hsa-miR-5787	hsa-miR-1273h-5p	hsa-miR-6884-5p
*hsa_circRNA_000479*	0.04	0.34	2.39	up	hsa-miR-942-5p	hsa-miR-4761-5p	hsa-miR-4753-3p	hsa-miR-4325	hsa-miR-6739-3p
*hsa_circRNA_060455*	<0.01	0.04	2.34	up	hsa-miR-4267	hsa-miR-4322	hsa-miR-4265	hsa-miR-1256	hsa-miR-599
*hsa_circRNA_102745*	<0.01	0.16	2.27	up	hsa-miR-5584-5p	hsa-miR-6797-5p	hsa-miR-4261	hsa-miR-147b-5p	hsa-miR-1249-5p
*hsa_circRNA_000897*	0.04	0.35	2.22	up	hsa-miR-6881-3p	hsa-miR-2355-3p	hsa-miR-6891-3p	hsa-miR-5193	hsa-miR-6740-3p
*hsa_circRNA_405788*	<0.01	<0.01	11.73	down	hsa-miR-6737-3p	hsa-miR-7152-5p	hsa-miR-4632-3p	hsa-miR-6748-3p	hsa-miR-941
*hsa_circRNA_104982*	<0.01	<0.01	10.38	down	hsa-miR-103a-1-5p	hsa-miR-103a-2-5p	hsa-miR-4778-3p	hsa-miR-1976	hsa-miR-483-3p
*hsa_circRNA_002008*	<0.01	<0.01	10.10	down	hsa-miR-627-3p	hsa-miR-6738-3p	hsa-miR-6781-3p	hsa-miR-5001-3p	hsa-miR-130a-5p
*hsa_circRNA_406768*	<0.01	<0.01	9.81	down	hsa-miR-4660	hsa-miR-500a-3p	hsa-miR-22-5p	hsa-miR-1908-3p	hsa-miR-4786-3p
*hsa_circRNA_037139*	<0.01	0.05	9.49	down	hsa-miR-6825-5p	hsa-miR-34a-5p	hsa-miR-4739	hsa-miR-34b-5p	hsa-miR-4690-5p
*hsa_circRNA_404686*	<0.01	0.06	8.69	down	hsa-miR-1249-5p	hsa-miR-3916	hsa-miR-6873-5p	hsa-miR-6760-5p	hsa-miR-4739
*hsa_circRNA_401844*	<0.01	0.07	8.25	down	hsa-miR-6818-5p	hsa-miR-8059	hsa-miR-4471	hsa-miR-612	hsa-miR-920
*hsa_circRNA_001882*	<0.01	<0.01	7.85	down	hsa-miR-1273h-5p	hsa-miR-619-5p	hsa-miR-6780a-5p	hsa-miR-30b-3p	hsa-miR-6884-5p
*hsa_circRNA_028882*	<0.01	<0.01	7.67	down	hsa-miR-4726-3p	hsa-miR-6078	hsa-miR-6868-3p	hsa-miR-608	hsa-miR-1538
*hsa_circRNA_065656*	<0.01	<0.01	7.17	down	hsa-miR-4449	hsa-miR-10398-3p	hsa-miR-3184-5p	hsa-miR-298	hsa-miR-6807-5p

The 10 most up- and downregulated circRNAs with a fold change > 2 and a *p* value < 0.05 are shown. MREs, miRNA response elements; FDR, false discovery rate; FC, fold change.

**Table 2:** Statistically significant circRNAs between fatal CCHF patients and controls.

circRNA	*p*-Value	FDR	FC (abs)	Regulation	MRE1	MRE2	MRE3	MRE4	MRE5
*hsa_circRNA_011083*	0.04	0.16	12.95	up	hsa-miR-2277-5p	hsa-miR-1285-3p	hsa-miR-148a-5p	hsa-miR-3127-5p	hsa-miR-6874-3p
*hsa_circRNA_011084*	0.01	0.10	4.60	up	hsa-miR-3940-3p	hsa-miR-2277-5p	hsa-miR-1285-3p	hsa-miR-10398-3p	hsa-miR-5197-3p
*hsa_circRNA_102485*	<0.01	0.06	4.14	up	hsa-miR-10396b-5p	hsa-miR-1269a	hsa-miR-6132	hsa-miR-6787-5p	hsa-miR-103a-1-5p
*hsa_circRNA_029031*	0.02	0.12	3.34	up	hsa-miR-6844	hsa-miR-6730-5p	hsa-miR-4763-3p	hsa-miR-4768-3p	hsa-miR-3180-5p
*hsa_circRNA_001161*	<0.01	0.01	2.98	up	hsa-miR-6787-5p	hsa-miR-1908-5p	hsa-miR-6756-5p	hsa-miR-25-5p	hsa-miR-4653-3p
*hsa_circRNA_102431*	0.01	0.09	2.93	up	hsa-miR-6743-5p	hsa-miR-4688	hsa-miR-5194	hsa-miR-1914-3p	hsa-miR-6738-5p
*hsa_circRNA_033572*	0.02	0.14	2.92	up	hsa-miR-6774-5p	hsa-miR-661	hsa-miR-4260	hsa-miR-637	hsa-miR-1227-5p
*hsa_circRNA_104642*	0.04	0.17	2.89	up	hsa-miR-3689d	hsa-miR-7847-3p	hsa-miR-4443	hsa-miR-6851-5p	hsa-miR-6511a-5p
*hsa_circRNA_092545*	0.04	0.17	2.87	up	hsa-miR-670-3p	hsa-miR-130b-5p	hsa-miR-4694-3p	hsa-miR-1250-3p	hsa-miR-3153
*hsa_circRNA_102323*	0.02	0.12	2.78	up	hsa-miR-665	hsa-miR-3616-3p	hsa-miR-11181-3p	hsa-miR-765	hsa-miR-4779
*hsa_circRNA_037139*	<0.01	0.04	20.38	down	hsa-miR-6825-5p	hsa-miR-34a-5p	hsa-miR-4739	hsa-miR-34b-5p	hsa-miR-4690-5p
*hsa_circRNA_403874*	0.01	0.09	15.46	down	hsa-miR-5189-5p	hsa-miR-612	hsa-miR-6860	hsa-miR-3187-5p	hsa-miR-571
*hsa_circRNA_404163*	<0.01	0.03	11.77	down	hsa-miR-4756-3p	hsa-miR-520a-5p	hsa-miR-525-5p	hsa-miR-4677-5p	hsa-miR-599
*hsa_circRNA_406983*	<0.01	0.06	10.79	down	hsa-miR-6767-3p	hsa-miR-16-1-3p	hsa-miR-4795-3p	hsa-miR-4789-5p	hsa-miR-8485
*hsa_circRNA_028882*	<0.01	0.01	9.77	down	hsa-miR-4726-3p	hsa-miR-6078	hsa-miR-6868-3p	hsa-miR-608	hsa-miR-1538
*hsa_circRNA_406768*	<0.01	0.01	9.76	down	hsa-miR-4660	hsa-miR-500a-3p	hsa-miR-22-5p	hsa-miR-1908-3p	hsa-miR-4786-3p
*hsa_circRNA_001937*	0.01	0.12	9.20	down	hsa-miR-11399	hsa-miR-1178-5p	hsa-miR-4677-5p	hsa-miR-22-5p	hsa-miR-26b-3p
*hsa_circRNA_405788*	<0.01	0.05	9.18	down	hsa-miR-6737-3p	hsa-miR-7152-5p	hsa-miR-4632-3p	hsa-miR-6748-3p	hsa-miR-941
*hsa_circRNA_104982*	<0.01	0.04	9.05	down	hsa-miR-103a-1-5p	hsa-miR-103a-2-5p	hsa-miR-4778-3p	hsa-miR-1976	hsa-miR-483-3p
*hsa_circRNA_101319*	<0.01	0.03	8.33	down	hsa-miR-138-5p	hsa-miR-3085-5p	hsa-miR-764	hsa-miR-2115-5p	hsa-miR-4793-5p

The 10 most up- and downregulated circRNAs with a fold change > 2 and a *p* value < 0.05 are shown. MREs, miRNA response elements; FDR, false discovery rate; FC, fold change.

**Table 3:** Statistically significant circRNAs between fatal CCHF patients and nonfatal cases.

circRNA	*p*-Value	FDR	FC (abs)	Regulation	MRE1	MRE2	MRE3	MRE4	MRE5
*hsa_circRNA_007738*	0.03	0.22	11.49	up	hsa-miR-3681-3p	hsa-miR-12119	hsa-miR-627-3p	hsa-miR-3192-5p	hsa-miR-1277-5p
*hsa_circRNA_000250*	0.03	0.22	6.37	up	hsa-miR-2053	hsa-miR-7109-3p	hsa-miR-10399-3p	hsa-miR-153-5p	hsa-miR-514b-5p
*hsa_circRNA_406281*	0.02	0.20	4.53	up	hsa-miR-7843-5p	hsa-miR-4436a	hsa-miR-5000-3p	hsa-miR-744-3p	hsa-miR-3659
*hsa_circRNA_102485*	0.01	0.19	4.20	up	hsa-miR-10396b-5p	hsa-miR-1269a	hsa-miR-6132	hsa-miR-6787-5p	hsa-miR-103a-1-5p
*hsa_circRNA_406111*	0.04	0.23	3.27	up	hsa-miR-4739	hsa-miR-6876-5p	hsa-miR-4728-5p	hsa-miR-4769-5p	hsa-miR-3153
*hsa_circRNA_044235*	0.03	0.21	3.20	up	hsa-miR-520f-3p	hsa-miR-4524a-5p	hsa-miR-4524b-5p	hsa-miR-636	hsa-miR-5590-5p
*hsa_circRNA_400037*	0.04	0.23	3.19	up	hsa-miR-6856-5p	hsa-miR-3173-3p	hsa-miR-4726-5p	hsa-miR-4751	hsa-miR-3154
*hsa_circRNA_102101*	0.01	0.20	3.09	up	hsa-miR-103a-1-5p	hsa-miR-103a-2-5p	hsa-miR-338-5p	hsa-miR-6815-3p	hsa-miR-4659a-3p
*hsa_circRNA_001161*	<0.01	0.18	3.06	up	hsa-miR-6787-5p	hsa-miR-1908-5p	hsa-miR-6756-5p	hsa-miR-25-5p	hsa-miR-4653-3p
*hsa_circRNA_405974*	0.03	0.21	3.03	up	hsa-miR-4726-3p	hsa-miR-4478	hsa-miR-4723-3p	hsa-miR-3929	hsa-miR-7111-3p
*hsa_circRNA_403874*	0.01	0.19	9.35	down	hsa-miR-5189-5p	hsa-miR-612	hsa-miR-6860	hsa-miR-3187-5p	hsa-miR-571
*hsa_circRNA_001937*	0.01	0.18	8.06	down	hsa-miR-11399	hsa-miR-1178-5p	hsa-miR-4677-5p	hsa-miR-22-5p	hsa-miR-26b-3p
*hsa_circRNA_000102*	0.04	0.23	6.90	down	hsa-miR-4796-5p	hsa-miR-11399	hsa-miR-4753-3p	hsa-miR-6873-3p	hsa-miR-1296-3p
*hsa_circRNA_402094*	0.02	0.20	6.78	down	hsa-miR-6860	hsa-miR-12121	hsa-miR-612	hsa-miR-670-5p	hsa-miR-3140-5p
*hsa_circRNA_403560*	0.03	0.22	6.76	down	hsa-miR-27b-3p	hsa-miR-27a-3p	hsa-miR-216a-3p	hsa-miR-4793-3p	hsa-miR-128-3p
*hsa_circRNA_103108*	0.02	0.20	5.80	down	hsa-miR-12115	hsa-miR-5089-5p	hsa-miR-654-5p	hsa-miR-541-3p	hsa-miR-6880-5p
*hsa_circRNA_030448*	0.03	0.22	5.75	down	hsa-miR-942-5p	hsa-miR-6809-3p	hsa-miR-4659b-3p	hsa-miR-4753-3p	hsa-miR-4659a-3p
*hsa_circRNA_102690*	<0.01	0.18	5.60	down	hsa-miR-2115-5p	hsa-miR-12124	hsa-miR-513b-3p	hsa-miR-5093	hsa-miR-3173-3p
*hsa_circRNA_400862*	<0.01	0.18	5.36	down	hsa-miR-1305	hsa-miR-650	hsa-miR-370-5p	hsa-miR-6868-3p	hsa-miR-1237-3p
*hsa_circRNA_002635*	0.01	0.19	5.30	down	hsa-miR-1255b-2-3p	hsa-miR-4786-3p	hsa-miR-4753-3p	hsa-miR-6715a-3p	hsa-miR-4782-5p

The 10 most up- and downregulated circRNAs with a fold change > 2 and a *p* value < 0.05 are shown. MREs, miRNA response elements; FDR, false discovery rate; FC, fold change.

## Data Availability

The original contributions presented in this study are included in the article/Supplementary Materials.

## Supplementary Materials

The following supporting information can be downloaded at: https://www.mdpi.com/article/10.3390/ijms27156738/s1.

## Abbreviations

CCHF	Crimean–Congo hemorrhagic fever
circRNAs	Circular RNAs
RT-PCR	Reverse transcription polymerase chain reaction
GO	Gene ontology
BP	Biological process
CC	Cellular component
MF	Molecular function
KEGG	Kyoto Encyclopedia of Genes and Genomes
CCHFV	Crimean–Congo hemorrhagic fever virus
cRNA	Complement RNA
miRNA	MicroRNA
rRNA	Ribosomal RNA
tRNA	Transfer RNA
dsRNA	Double-stranded RNA
mRNA	Messenger RNA
FDR	False discovery rate
FC	Fold change
